# Nup153 is not required for anchoring heterochromatic DSBs to the nuclear periphery

**DOI:** 10.17912/micropub.biology.001176

**Published:** 2024-04-26

**Authors:** Taehyun Ryu, Chiara Merigliano, Irene Chiolo

**Affiliations:** 1 Molecular and Computational Biology Department, University of Southern California, Los Angeles, CA, USA; 2 Department of Genetics, Harvard Medical School, Boston, MA, USA

## Abstract

Pericentromeric heterochromatin mostly comprises repeated DNA sequences prone to ectopic recombination. In *Drosophila* cells, ‘safe’ homologous recombination repair requires relocalization of heterochromatic repair sites to the nuclear periphery before Rad51 recruitment and strand invasion. DSBs are anchored to the nuclear periphery through the Nup107/160 nucleoporin complex. Previous studies suggested that the nuclear pore ‘basket’ protein Nup153 could also mediate anchoring, but Nup153 RNAi depletion also affects Nup107 association with the pores, preventing a direct assessment of Nup153 role. Using a separation of function mutant, here we show that Nup153 is not required for anchoring heterochromatic DSBs to the nuclear periphery.

**Figure 1. Nup153 DNA-binding and FG-repeat domains are not required for relocalization of heterochromatic DSBs.**
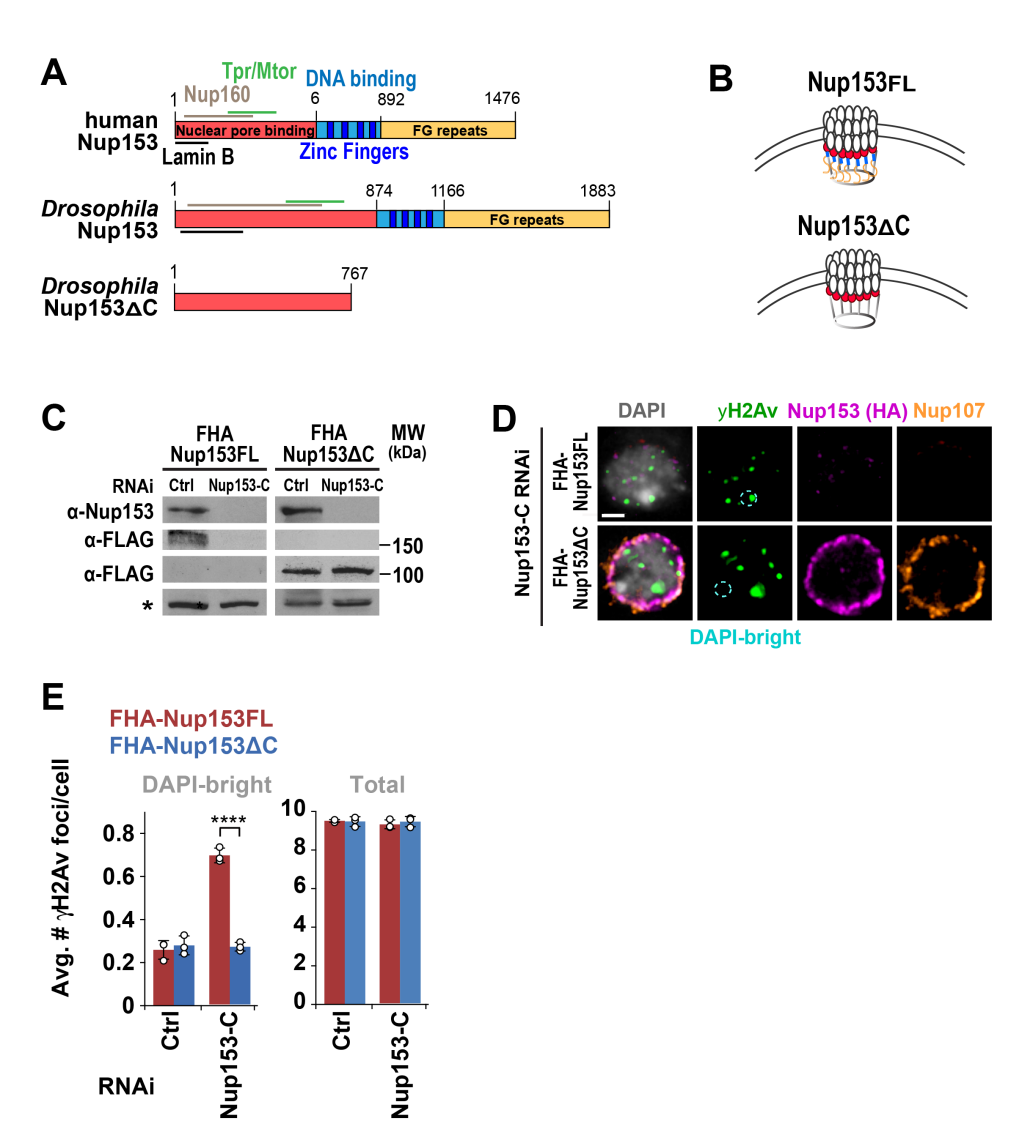
**A:** Scheme of human and *Drosophila* Nup153 protein, highlighting the domains mapped in the human protein and conserved domains in the *Drosophila* protein. The N-terminus is responsible for binding other pore proteins and Lamina B, as indicated. **B:** Schematic representation of Nup153 FL and ΔC at the pore. **C:** Western blot analysis with the indicated antibodies of cells expressing FLAG-HA(FHA)-Nup153FL and FHA-Nup153ΔC shows efficient depletion of endogenous Nup153 and FHA-Nup153FL. (*) A non-specific band is used as a loading control. **D:** Immunofluorescence (IF) of indicated proteins in cells expressing FHA-Nup153FL or FHA-Nup153ΔC after RNAi depletion of endogenous Nup153 and FHA-Nup153FL. Scale bar = 1µm. **E:** Quantification of γH2Av foci in DAPI-bright and total foci in cells expressing FHA-Nup153FL or FHA-Nup153ΔC fixed 60 min after IR, following indicated RNAi depletions. Nup153-C indicates that siRNAs recognize the C-terminal region of the protein. ****P < 0.0001, two-tailed Mann-Whitney test, n > 166 cells from three independent experiments. Error bars: mean +/- SD.

## Description

Pericentromeric heterochromatin (hereafter "heterochromatin") constitutes about 30% and 10% of fly and human genomes respectively, and is mostly composed of repeated DNA sequences (Lander et al. 2001, Hoskins et al. 2007, Ho et al. 2014, Hoskins et al. 2015, Nurk et al. 2022) . In heterochromatin, thousands to millions of identical DNA repeats, including from different chromosomes, can engage in ectopic recombination (Peng and Karpen 2008, Amaral et al. 2017) , presenting a serious threat to genome stability in multicellular eukaryotes. In *Drosophila* , heterochromatin forms a distinct nuclear domain (Chiolo et al. 2011, Riddle et al. 2011, Li et al. 2017, Caridi et al. 2018, See et al. 2021) and aberrant recombination is prevented by relocalization of double-strand breaks (DSBs) to the nuclear periphery before Rad51 recruitment and strand invasion (Chiolo et al. 2011, Ryu et al. 2015, Ryu et al. 2016, Caridi et al. 2018, Merigliano et al. 2023, Miller et al. 2023) . Early HR steps occur within the heterochromatin domain, while later steps are initially blocked and resume only after relocalization (Chiolo et al. 2011, Ryu et al. 2015, Ryu et al. 2016, Caridi et al. 2018, Merigliano et al. 2023) .

This movement relies on the SUMO E3 ligase Smc5/6 complex (Chiolo et al. 2011, Ryu et al. 2015, Ryu et al. 2016) and its interactors: the actin nucleator Arp2/3 (Caridi et al. 2018) and nuclear myosins (Caridi et al. 2018) . Arp2/3 coordinates the formation of nuclear actin filaments that start polymerizing at heterochromatic DSBs and enable the directed, myosin-driven, movement of repair sites to the nuclear periphery (Caridi et al. 2018) . The mobilization of heterochromatic repair sites also requires nucleoporins associated with the chromatin in the nucleoplasm, particularly the phase separation properties of Nup98 (Merigliano et al. 2023) . Nup98 creates a condensate inside the heterochromatin domain, facilitating the initial diffusive movement of repair sites from the core to the periphery of the heterochromatin domain, where capturing from nuclear F-actin occurs (Merigliano et al. 2023) . Once at the nuclear periphery, repair sites associate with the nuclear pores through their interaction with the Y complex, including Nup107 and Nup160 (Ryu et al. 2015) . These might enable repair restart through the degradation of SUMOylated targets mediated by the SUMO-targeted ubiquitin ligase (STUbL) Dgrn (Ryu et al. 2015) .

Relocalization likely prevents aberrant recombination by separating damaged DNAs from ectopic repeated sequences, thus promoting ‘safe’ exchanges with the sister chromatid or the homologous chromosome (Caridi et al. 2017, Caridi et al. 2019, Rawal et al. 2019) . Accordingly, loss of components required for relocalization results in persistent unrepaired heterochromatic DSBs and widespread chromosome rearrangements (Chiolo et al. 2011, Ryu et al. 2015, Ryu et al. 2016, Caridi et al. 2018, Dialynas et al. 2019, Merigliano et al. 2023) . Similar mechanisms have been described in mammalian cells (Jakob et al. 2011, Chiolo et al. 2013, Tsouroula et al. 2016, Caridi et al. 2018) , revealing conserved pathways.

In *Drosophila* cells, repair sites leave the heterochromatin domain 10 min after DSB induction with ionizing radiation (IR). This results in fewer repair sites (γH2Av foci) in DAPI-bright heterochromatin (a subsection of the heterochromatin domain (Chiolo et al. 2011) ) 60 min after IR (Chiolo et al. 2011, Ryu et al. 2015, Caridi et al. 2018, Merigliano et al. 2023) . In the absence of Nup107, defective anchoring to the nuclear periphery results in a higher number of γH2Av foci in DAPI-bright 60 min after IR, without affecting total focus count (Ryu et al. 2015) . A similar result is observed after RNAi depletion of the nuclear pore basket protein Nup153 (Ryu et al. 2015) . However, Nup153 RNAi also results in significant loss of the nuclear periphery signal of Nup107 (Ryu et al. 2015) , consistent with a role for Nup153 in nuclear pore assembly and Nup107 recruitment (Vollmer et al. 2015) . Thus, the effect of Nup153 RNAi on relocalization of heterochromatic DSBs could be indirect, through the loss of Nup107 (Ryu et al. 2015) .

Here we assessed the role of Nup153 in heterochromatin repair by expressing in *Drosophila* cells a Nup153∆C mutant, which retains the N-terminal domain required for Nup107 recruitment while losing DNA binding and pore transport domains (Griffis et al. 2004) ( Figure 1A,B ). As a control, we generated cells expressing Nup153 full-length (FL). We RNAi depleted Nup153 by targeting its C-terminal region (Nup153-C RNAi), which affects the levels of endogenous Nup153 and FHA-Nup153FL, without altering FHA-Nup153∆C ( Figure 1C ). As expected, RNAi depletion of Nup153 results in significant loss of Nup107 signal at the nuclear periphery ( Figure 1D ) (Ryu et al. 2015) . However, cells display normal nuclear size and decondensed chromatin ( Figure 1D ), suggesting that other components of the pore are intact (Boehmer et al. 2003) or that residual Nup107 is present to sustain some pore assembly (Vollmer et al. 2015) . Importantly, expression of Nup153∆C in cells depleted for endogenous Nup153 fully restores Nup107 signal at the pores ( Figure 1D ), consistent with the Y complex being intact.

Next, we tested how the loss of most Nup153 domains affects heterochromatin repair by investigating the distribution of repair foci relative to DAPI-bright in cells expressing Nup153∆C. RNAi depletion of endogenous Nup153 and FHA-Nup153FL results in a higher number of γH2Av foci in DAPI-bright heterochromatin 60 min after IR, consistent with a relocalization defect and previous studies (Ryu et al. 2015) . However, expression of FHA-Nup153∆C in the absence of endogenous Nup153 lowers the number of γH2Av foci in DAPI-bright 60 min after IR to a level similar to that observed in control RNAi cells, without affecting total focus count ( Figure 1D ). This is consistent with normal relocalization of heterochromatic DSBs. Thus, focus relocalization occurs normally in cells lacking all the N-terminal Nup153 functional domains, including DNA-binding activities and FG repeats. We conclude that Nup153 is not required for relocalizing heterochromatic DSBs, and thus it does not contribute to anchoring heterochromatic repair sites to the nuclear periphery.

## Methods

**Cell cultures**

Kc167 (Kc) cells were used for all experiments and were maintained as logarithmically growing cultures in Schneider’s medium (Sigma) or Sf-900 II (ThermoFisher) + 10% FBS (GemCell US Origin, Gemini) + antibiotic-antimycotic (ThermoFisher). Kc cells were authenticated by the Drosophila Genomic Resource Center (DGRC) and no mycoplasma contamination was detected.

**Generation of cell lines expressing tagged proteins**

Experiments were performed using stable cell lines obtained by cotransfecting each plasmid of interest with pCoHygro (Invitrogen) and selecting in the presence of 100 μg/ml Hygromycin B (Enzo Life Sciences) for ~1 month. Transfections were performed with Cellfectin (Life Technologies), according to manufacturers’ procedures.

**IR Treatments**

Cultures were exposed to IR using a 160 kV X-ray source (X-RAD iR-160, Precision X-Ray). A dose of 5 Gy was used for the experiment, as in previous studies (Chiolo et al. 2011, Ryu et al. 2015, Ryu et al. 2016, Caridi et al. 2018, Merigliano et al. 2023) .

**Plasmids**

pCopia-3xFlag-3xHA(FHA)-Nup153 FL or ∆C plasmids were generated by inserting the corresponding PCR-amplified sequence of Nup153 into a pCopia-FHA backbone (Chiolo et al. 2011, Ryu et al. 2015, Ryu et al. 2016, Caridi et al. 2018, Merigliano et al. 2023) , after AscI/PacI digestion. The cDNA template used was LD46479 from DGRC.

**dsRNA synthesis and sequences**

dsRNAs were prepared with the MEGAscript T7 Kit (Thermo Fisher Scientific Cat# AM1334). dsRNAs targeting the C-terminus of Nup153 were prepared using the oligos: Nup153 for: 5’-CTAATACGACTCACTATAGGGAG-CCCACACCTTTGTCGAACTT and Nup153 rev: 5’-CTAATACGACTCACTATAGGGAG-ACAGGCCACACACTTGTTGA.

**RNAi depletions in cultured cells**

dsRNAs were transfected with DOTAP Liposomal Transfection Reagent (Roche) following manufacturer’s instructions. Incubation times and dsRNA amounts were optimized to maximize depletion efficiency while avoiding toxicity and cell cycle effects. Cells were treated with dsRNAs for 5 days For Nup153 depletion. The control (Ctrl) used for all RNAi experiments is RNAi depletion of the brown (bw) gene transcript, which regulates the body color of adults flies and is not involved in DNA repair pathways (Chiolo et al. 2011, Ryu et al. 2015, Caridi et al. 2018, Merigliano et al. 2023) .

**Western blotting**

1-3×10 ^6^ cells were collected, washed once in PBS and lysed for 15–20 min on ice with lysis buffer (50 mM Tris, pH 7.8, 1% NP-40, 150 mM NaCl) containing protease inhibitors (Complete, Roche), 2-mercaptoethanol, and 1 mM PMSF. Benzonase was added to each sample (0.5 U/ul) for 30 min. The soluble lysate was recovered by centrifugation (10 min, 4°C) and resuspended in loading buffer (Laemmli) to a final concentration of 1x. Samples were denatured for 5 min at 95°C before running them on a TGX 4–12% gel (Bio-Rad). Samples were then transferred onto nitrocellulose membrane for hybridization with specific antibodies.

**Immunofluorescence and quantification of repair foci in fixed samples**

Immunofluorescence was performed as previously described (Chiolo et al. 2011, Ryu et al. 2015, Caridi et al. 2018, Merigliano et al. 2023) . Imaging and image processing was performed with the DeltaVision Deconvolution microscope and the Softworx Software as previously described (Chiolo et al. 2011, Ryu et al. 2015, Caridi et al. 2018, Merigliano et al. 2023) . Classification of repair foci inside or outside the DAPI-bright region was done as in (Chiolo et al. 2011, Ryu et al. 2015, Caridi et al. 2018, Merigliano et al. 2023) .

**Statistical analysis**

All statistical analyses were performed using Prism6 (Graphpad Software).

## Reagents

**Table:** 

**Antibodies**			
**Name**	**concentration**	**source**	**catalogue number**
Histone H2AvD phosphoS137	1:500 IF	Rockland	600-401-914
anti-Nup153	1:500 WB	M. Capelson	N/A
anti-Nup107	1:2000	V. Doye	N/A
anti-HA	1:1000 IF	DSHB	rMs-IgG1
anti-Flag	1:1000 WB	Sigma-Aldrich	F1804

IF: immunofluorescence, WB: western blotting, DSHB: Developmental Studies Hybridoma Bank.

**Table:** 

**Plasmids name**	**Source**	**Stock #**
pCopia-FHA-Nup153	This study	p155
pCopia-FHA-Nup153∆C	This study	p394
